# Clinical value of next generation sequencing of plasma cell-free DNA in gastrointestinal stromal tumors

**DOI:** 10.1186/s12885-020-6597-x

**Published:** 2020-02-05

**Authors:** César Serrano, Ana Vivancos, Antonio López-Pousa, Judit Matito, Francesco M. Mancuso, Claudia Valverde, Sergi Quiroga, Stefania Landolfi, Sandra Castro, Cristina Dopazo, Ana Sebio, Anna C. Virgili, María M. Menso, Javier Martín-Broto, Miriam Sansó, Alfonso García-Valverde, Jordi Rosell, Jonathan A. Fletcher, Suzanne George, Joan Carles, Joaquín Arribas

**Affiliations:** 1 Medical Oncology Department, Vall d’Hebron University Hospital, P. Vall d’Hebron 119, 08035 Barcelona, Spain; 2 Preclinical Research Program, Vall d’Hebron Institute of Oncology, Barcelona, Spain; 3 Cancer Genomics Group, |Vall d’Hebron Institute of Oncology, Natzaret 115, 08035 Barcelona, Spain; 4 Medical Oncology, Sant Pau University Hospital, Barcelona, Spain; 5 Radiology Department, Vall d’Hebron University Hospital, Barcelona, Spain; 6 Pathology Department, Vall d’Hebron University Hospital, Barcelona, Spain; 7 Surgical Oncology Division, Vall d’Hebron University Hospital, Barcelona, Spain; 8 Radiology Department, Sant Pau University Hospital, Barcelona, Spain; 9 Medical Oncology, Virgen del Rocío Hospital, Sevilla, Spain; 10 Pathology Department, Brigham and Women’s Hospital/Harvard Medical School, Boston, USA; 11 Center for Sarcoma and Bone Oncology, Dana-Farber Cancer Institute, Boston, USA; 12 Institució Catalana de Recerca i Estudis Avançats (ICREA), Barcelona, Spain

**Keywords:** Circulating tumor DNA, Gastrointestinal stromal tumor, Imatinib, KIT, Liquid biopsy, PDGFRA, Sarcoma, Regorafenib, Sunitinib

## Abstract

**Background:**

Gastrointestinal stromal tumor (GIST) initiation and evolution is commonly framed by KIT/PDGFRA oncogenic activation, and in later stages by the polyclonal expansion of resistant subpopulations harboring KIT secondary mutations after the onset of imatinib resistance. Thus, circulating tumor (ct)DNA determination is expected to be an informative non-invasive dynamic biomarker in GIST patients.

**Methods:**

We performed amplicon-based next-generation sequencing (NGS) across 60 clinically relevant genes in 37 plasma samples from 18 GIST patients collected prospectively. ctDNA alterations were compared with NGS of matched tumor tissue samples (obtained either simultaneously or at the time of diagnosis) and cross-validated with droplet digital PCR (ddPCR).

**Results:**

We were able to identify cfDNA mutations in five out of 18 patients had detectable in at least one timepoint. Overall, NGS sensitivity for detection of cell-free (cf)DNA mutations in plasma was 28.6%, showing high concordance with ddPCR confirmation. We found that GIST had relatively low ctDNA shedding, and mutations were at low allele frequencies. ctDNA was detected only in GIST patients with advanced disease after imatinib failure, predicting tumor dynamics in serial monitoring. KIT secondary mutations were the only mechanism of resistance found across 10 imatinib-resistant GIST patients progressing to sunitinib or regorafenib.

**Conclusions:**

ctDNA evaluation with amplicon-based NGS detects KIT primary and secondary mutations in metastatic GIST patients, particularly after imatinib progression. GIST exhibits low ctDNA shedding, but ctDNA monitoring, when positive, reflects tumor dynamics.

## Background

Gastrointestinal stromal tumor (GIST) is a rare cancer of mesenchymal origin, with an incidence rate of approximately 1 case/100,000/year [1, 2]. Oncogenic activation of KIT or PDGFRA receptor tyrosine kinases (RTKs) is central to GIST biology, and are present in 85–90% of the patients [3, 4]. Specifically, two thirds of GISTs harbor a wide array of primary mutations in *KIT* juxtamembrane domain, encoded by exon 11. Similar complexity is found in other *KIT* regions (exons 9, 13 and 17) [5]. Likewise, mutually exclusive primary mutations in PDGFRA are found in homologous regions [6]. Although most advanced GISTs respond to first-line inhibitor imatinib [7], disease progression eventually occurs in 20–24 months after treatment initiation. Acquired resistance to imatinib is due in 70–90% of GIST patients to the expansion of subpopulations harboring different KIT secondary mutations [8–10] that cluster in the ATP-binding pocket and the activation loop [5, 8–10]. Resistance mechanisms after several lines of treatments are yet to be fully elucidated [11].

Importantly, KIT/PDGFRA primary and secondary genotype is relevant for GIST clinical management because it predicts GIST clinical behavior and efficacy from tyrosine kinase inhibitors (TKIs) with KIT inhibitory activity in the first line [12] – imatinib – and in any line of treatment after imatinib failure, including standard second- (sunitinib) and third-line treatments (regorafenib) [13–17]. Therefore, detection and monitoring of GIST primary and resistance mutations in circulating tumor DNA (ctDNA) has the potential to improve molecular profiling, surveillance and treatment decision-making.

qPCR or digital PCR-based technologies have the highest analytical sensitivity for mutation detection [18–20]. While PCR plasma genotyping is preferred for recurrent predictable aberrations, technologies based on next-generation sequencing (NGS) have the potential to asses more broadly the variety of primary and resistance mutations [21–23]. Thus, the complexity and diversity of KIT primary and secondary mutations in imatinib-sensitive and –resistant patients favors the use of NGS over PCR for the detection of cfDNA mutations. NGS technologies employ various strategies for enriching specific target regions, and some of them are commercially available for their use in plasma [24, 25]. By contrast, amplicon-based target enrichment, although less sensitive, has a widespread use in molecular screening programs using tumor tissue, and it is progressively emerging as an alternative approach for extensive cfDNA assessment [26, 27]. This, in turn, would potentially facilitate the implementation of cfDNA evaluation in oncology centers with expertise in NGS.

Overall, there is an urgent need for real-time tumor biomarkers to guide therapy selection in GIST. Nevertheless, until ctDNA is proven to render the genomic information detected in solid tissue, it cannot replace the need for metastatic tissue biopsy of patients, nor guide clinical decisions [28]. To address this, we orthogonally validated an amplicon-based NGS panel for routine molecular prescreening in a cohort of localized and advanced GIST patients with matched tissue and plasma samples, and in a second cohort with serial plasma determinations.

## Methods

### GIST patient cohorts

Localized and metastatic KIT- or PDGFRA-mutant GIST patients were prospectively enrolled in a tissue and plasma acquisition protocol. Consenting patients were distributed in two cohorts: cohort A (matched tissue/plasma) included localized or metastatic GIST patients with matched tissue and plasma samples obtained simultaneously. Localized patients were imatinib-naïve and tissue samples were obtained through surgical removal of the primary tumors. Tumor tissue in metastatic patients included resections of unifocal progressive disease. In all cases plasma samples were collected 7 to 14 days before tumor resection. Plasma samples from patients on TKI treatment were obtained while on drug. Cohort B (serial) included metastatic patients with serial plasma samples throughout the course of their treatment, together with tumor tissue at the time of diagnosis.

This study was approved by the Institutional Review Board from each participating center and written informed consent was obtained from all patients to donate blood samples and tumor tissue.

### Blood sample collection and plasma processing

Peripheral blood was collected into EDTA tubes (Beckton Dickinson) and plasma was extracted within 4 h of blood collection through two centrifugation steps of 10 min each, the first at 1600 g and the second at 3000 g. Single-use 1.5 mL plasma aliquots were obtained and stored at − 80 °C until use. cfDNA was obtained from 3 mL of plasma using the QIAamp Circulating Nucleic Acids kit (Qiagen) and quantified with a Qubit Fluorometer (ThermoFisher Scientific).

### Tumor tissue specimen collection and processing

Representative formalin fixed, paraffin-embedded (FFPE) tumor tissue blocks were retrieved from each case and reviewed by a GIST expert pathologist (S.L.). Five 10-μm tissue sections with more than 20% tumor area were obtained. DNA extraction was performed with the automated system Maxwell16 FFPE plus LEV DNA purification kit (Promega). DNA quality and concentration were measured with a NanoDrop 1000 spectrophotometer (Thermo Scientific, Waltham, MA).

In order to optimize VHIO amplicon-sequencing pipeline, seven additional FFPE primary tumor samples were retrieved from our GIST series database with known *KIT* exon 11 long insertions and/or deletions (indels) (> 15 base pairs) through Sanger Sequencing [29].

### DNA from GIST cell lines

DNA from two human GIST cell lines with known long *KIT* exon 11 in-frame deletions was also used to optimize the NGS pipeline for the detection of long indels (> 15 base pairs). GIST-T1 has deleted 19 aminoacids (*KIT* exon 11 p.V560_Y578del), and GIST430 has 17 (*KIT* exon 11 p.V560_ L576del) [30].

### Tumor and plasma mutational analysis by amplicon sequencing

An initial multiplex-PCR with a proof-reading polymerase was performed on all samples. Tumor and plasma DNA were sequenced with an in-house developed amplicon-sequencing panel of over 1330 primer pairs targeting frequent mutations in oncogenes and several tumor suppressors, totaling 60 genes (Additional file 1: Table S1) including KIT and PDGFRA, which contain reported hotspots for primary and secondary mutations in GIST [31]. 500 ng of DNA from each tissue sample, or total cfDNA from 3 mL plasma samples were used for library preparation according to our established protocols. Duplicate chemistries were performed for each sample. Plasma analyses were carried out blinded to clinical information such as tumor genotype.

Amplicon sequencing was performed as previously described [31–33]. Specifically for this study, indexed libraries were pooled and sequenced in a HiSeq 2500 instrument (2X100) at an average coverage of 1000x for tissues samples and 5000x for plasmas. Variants were called using VarScan2 (v2.3.9) with the following parameters: minimum variant allele frequency (VAF) of 3% for FFPE samples and 1% for plasma samples; total coverage ≥10 reads; variant coverage ≥7 reads, and a *p*-value < 0.05. Germline mutations were manually excluded.

Recent studies revealed shortcomings of state-of-the-art variant callers that might fail to detect complex indels [34]. For this reason, an alternative pipeline for the detection of large indels in *KIT* exon 11 was also used. Filtered reads were re-mapped using bwa with relaxed values in parameters for long gaps: increasing maximum number of gap extensions (−e) and the maximum occurrences for extending a long deletion (−d), and decreasing the penalties for opening (−O) or extending a gap (−E). The resulting SAM file was used as input for the indel caller SOAPindel.

### Droplet digital PCR (ddPCR)

The QX200 ddPCR System (Bio-Rad Laboratories, Hercules, CA) was used to confirm in plasma the presence of variants detected in tumor tissue and plasma by amplicon sequencing. Genomic DNA from tumor tissue from the same patient was run as positive control. Custom Taqman SNP genotyping assays for ddPCR were designed to detect KIT and PDGFRA mutations (Additional file 2: Table S2). 1.5 mL of plasma was used for ddPCR validations.

Briefly, the 20 μL final volume of TaqMan PCR reaction mixture was assembled with 1x ddPCR Supermix for Probes (no dUTP), 900 nM of each primer, 250 nM of each probe and 8 μL of cfDNA or 30 ng per reaction in FFPE (positive controls). Each assay was performed in triplicate in separate mixes and loaded in different wells for amplification. The thermal cycling program was performed according to specifications of the manufacturer. After PCR, droplets were read in the Droplet Reader and analyzed with QuantaSoft version 1.7.4. Human reference genomic DNA was included as negative control and used to determine the cutoff for allele calling in each assay. ddPCR validations were carried out blinded to tumor genotype information.

### Statistical analysis

Descriptive statistics were used to characterize patients at study entry. Fisher’s exact test, Mantel-Haenzel test and Mann-Whitney test were used, depending on each variable type, to determine the association between clinicopathological and molecular features with detection of ctDNA. Concordance of VAFs between plasma ddPCR and NGS was calculated using Pearson Correlation. All statistical tests were conducted at a 2-sided significance level of 0.05. Analyses were performed using GraphPad Prism 6 (GraphPad Software, Inc., La Jolla, CA) or IBM SPSS Statistics 20.0 (Chicago, IL).

## Results

### Clinical patient cohorts

**Fig. 1:**
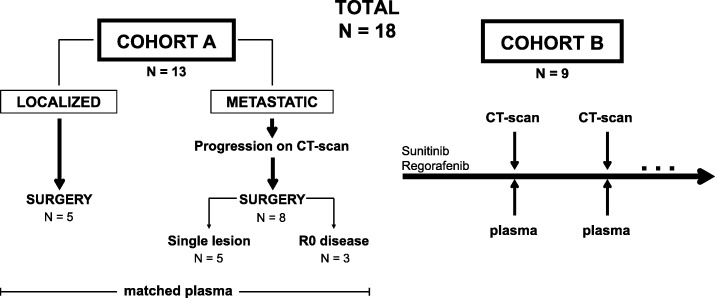
Schematic view of the study design and analyzed cases. Cohort A: matched tissue and plasma samples collected simultaneously. Cohort B: serial plasma monitoring in imatinib-resistant GIST patients

The current study recruited prospectively 18 KIT- or PDGFRA-mutant GIST patients from July 2015 to December 2017. Figure 1 displays an overview of both patient cohorts. Cohort A (matched tissue/plasma) included 13 localized (*n* = 5) or metastatic (*n* = 8) patients with matched tissue and plasma samples collected simultaneously. Matched tissue in metastatic patients included resections of a single progressing lesion (n = 5) or surgery of unifocal progressive disease aiming R0 disease (*n* = 3). Cohort B (serial) included nine imatinib-resistant, metastatic GIST patients treated with sunitinib or regorafenib in which serial plasma samples were collected simultaneously to CT-scans throughout the clinical course of the disease. These nine patients include four from cohort A that also had serial plasma determinations. Patients in cohort B had tumor tissue available, either at diagnosis or through tumor resection of metastatic disease (cohort A).

Clinicopathological and molecular features from all GIST patients included in this study are shown in Table 1.

**Table 1:** Patient demographics and disease characteristics (*N* = 18)

Characteristics	No. of Patients	%
Median age (range), y	64 (34–79)	N.A.
Sex		
Male	13	72.2
Female	5	27.8
Primary tumor location		
Stomach	8	44.4
Small bowel	7	38.9
Other	3	16.7
Median tumor size (range), cm		
<5	4	23.5
5–10	5	29.4
>10	8	47.1
Median mitotic count (/50HPF)		
<5	6	42.8
5–10	4	28.6
>10	4	28.6
Primary mutation		
KIT exon 11	14	77.8
PDGFRA exon 12	2	11.1
PDGFRA exon 18	2	11.1
Tumor dissemination		
Localized	5	27.8
Metastatic	13	72.2
Metastases location (*n* = 13)		
Liver	9	69.2
Peritoneum	10	77.0
Other	4	30.8
Median tumor burden metastatic disease, mm (range)^a^	152 (26–289)	N.A.
Sensitivity to imatinib		
Sensitive	7	38.8
Resistant	11	63.2

^a^Median tumor burden has been calculated as measurable disease at the initiation of treatment by RECIST criteria. Abbreviations: *No.* number, *y* years, *N.A.* not applicable, *cm* centimeters, *HPF* high-power fields, *mm* millimeters, *TKI* tyrosine kinase inhibitor

### Detection of long indels in *KIT* exon 11

An alternative pipeline for the detection of large indels in *KIT* exon 11 was evaluated. A total of nine cases, including two GIST cell lines, with known *KIT* exon 11 genotypes, were used to validate the alternative pipeline for the detection of large (> 15 base pairs) and/or complex indels in *KIT* exon 11.

Three out of nine cases were called with the standard pipeline while eight of nine cases could be identified with the optimized, alternative pipeline, therefore increasing the overall sensitivity of the NGS panel from 33.3 to 88.8% for the detection of large and/or complex indels in *KIT* exon 11 (Additional file 3: Table S3).

Analytical and clinical validity of amplicon-based NGS in the detection of ctDNA in GIST.

**Fig. 2:**
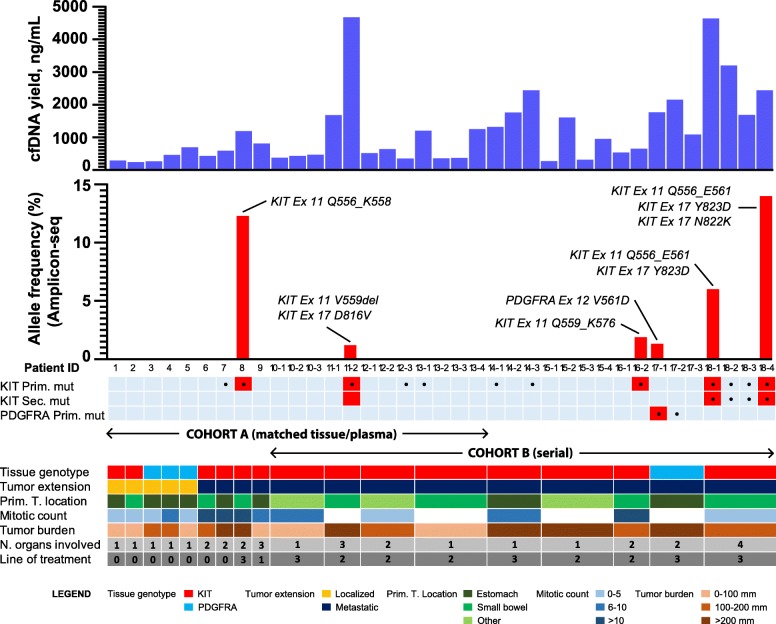
Schematic view showing the relation between cfDNA determined in plasma (ng/mL) and cases with ctDNA, totaling 37 plasma samples from 18 patients. cfDNA mutations detected by NGS are depicted in red bars with their corresponding AFs (%).The AF represented in the event of ≥2 mutations per sample is the highest. Samples from the same patient at different timepoints are represented using the patient number followed by a dash and the number of the timepoint. Genotype from cfDNA mutations has been divided accordingly to the type of mutation. Black spots in the KIT/PDGFRA genotype part of the graph represent positive samples for ddPCR. At the bottom, it is displayed an overview of clinical, pathological and molecular characteristics

Plasma samples and matched tumor tissue were obtained simultaneously in cohort A from a total of 13 KIT- or PDGFRA-mutant GIST patients, and were subjected to amplicon-sequencing of 60 cancer-related genes. All localized GIST were treatment-naïve. The eight metastatic GIST patients were either treatment naïve (*n* = 2) or progressing to first-to-third line TKIs at the time of tissue and plasma collection (Fig. 2, Additional file 4: Table S4). Only one of 13 cfDNA matched samples in cohort A (patient 8) had detectable ctDNA, with an allele fraction (AF) of 12.3%. The *KIT* exon 11 mutation detected in plasma matched with its respective mutation in tumor tissue. ctDNA was not detected in any of the five localized GIST before surgical resection, nor in the seven remaining metastatic patients at the timepoint of tumor tissue sample acquisition (Fig. 2). Three metastatic tissue samples harbored imatinib-resistance mutations, all of them emerging in the activation loop of KIT, encoded by *KIT* exon 17 and affecting codons N822K (patient 9), D820Y (patient 10) and D816V (patient 11) (Additional file 4: Table S4). No other resistance mutations were found by the NGS panel either in tissue or in plasma.

Cohort B consists of nine metastatic, imatinib-resistant GIST patients with serial plasma determinations, totaling 28 timepoints (Figs. 2, Additional file 4: Table S4). Four patients (44.4%) had ctDNA mutations in at least one timepoint, totaling five ctDNA-positive plasma samples (17.9%) (Fig. 2). Seven different mutations were found, all of them involving classical GIST drivers KIT and PDGFRA genes, with a median AF of 6.2% (range 1–14%). Primary mutations in *KIT* exon 11 (patients 11, 16 and 18) and *PDGFRA* exon 12 (patient 17) were detected across the four plasma samples. KIT secondary mutations were found in the two KIT-driven GIST patients, affecting exclusively *KIT* exon 17 at the codons D816V in patient 11, and N822K and Y823D in patient 18.

In order to determine the sensitivity of our amplicon-sequencing approach in plasma of GIST patients, tissue genotype was the reference standard, and ddPCR was performed for orthogonal validation. Sensitivity for detection of KIT/PDGFRA primary mutations and KIT secondary mutations in cfDNA was 28.6% (6/21). All but one mutation was confirmed by ddPCR. As expected, ddPCR had better sensitivity for plasma mutations than NGS, 42.9% (9/21) (Table 2). Quantitative concordance of AF between NGS and ddPCR for detection of plasma mutations was high across all plasma samples studied with both assays (*R*^*2*^ = 0.87) (Additional file 6: Figure S1A). Most discrepancies between NGS and ddPCR in plasma sequencing were related to variants with AF lower than NGS panel detection limit (<1%) (Additional file 6: Figure S1B and Table 2).

**Table 2:** Correlation of KIT/PDGFRA genotype between tissue and plasma

			cfDNA genotype (%AF)			
Patient ID	Cohort	Tissue genotype	Primary mut.		Resistance mut.	
			NGS	ddPCR	NGS	ddPCR
1	A	*KIT* W557R	0	0	N.A.	N.A.
2	A	*KIT* L576P	0	0	N.A.	N.A.
3	A	*PDGFRA* D842V	0	0	N.A.	N.A.
4	A	*PDGFRA* D842_D846	0	0	N.A.	N.A.
5	A	*PDGFRA* V561D	0	0	N.A.	N.A.
6	A	*KIT* Y568_L576	0	0	N.A.	N.A.
7	A	*KIT* K550_K558	0	1.5	N.A.	N.A.
8	A	*KIT* Q556_K558	12.3	15.2	N.A.	N.A.
9	A	*KIT* W557_K558 + N822K	0	0	0	0
10	A + B	*KIT* M552_E554 + D820Y	0	0	0	0
11	A + B	*KIT* V559del + D816V	1.2	0.5	1	0
12	A + B	*KIT* V555_V560	0	0.9	N.A.	N.A.
13	A + B	*KIT* T557_D572	0	1.2	N.A.	N.A.
14	B	*KIT* p.W557R	0	0.3	N.A.	N.A.
15	B	*KIT* V555_K558	0	0	N.A.	N.A.
16	B	*KIT* V559_L576	1.9	9.5	N.A.	N.A.
17	B	*PDGFRA* V561D	1.3	0.4	N.A.	N.A.
18	B	*KIT* Q556_E561	6	4.9	N.A.	N.A.

Abbreviations: *cfDNA* cell free DNA, *AF* allele frequency, *mut* mutation, *NGS* next generation sequencing, *ddPCR* droplet digital PCR, *N.A.* not applicable

Therefore, we observed good correlation between NGS and ddPCR for ctDNA detection in GIST. ddPCR was more sensitive than NGS, being most discrepancies observed at low AFs.

### Clinicopathological factors associated with ctDNA release in GIST

Baseline primary tumor characteristics such as age at diagnosis, primary tumor location, size, mitotic count and genotype, did not predict for the presence of ctDNA in plasma. Likewise, cfDNA load (median cfDNA concentration of 966.5 ng/mL, range 242–3200 ng/mL across 37 plasma samples) was not associated with ctDNA detection (*p* = 0.271). Conversely, only GIST patients with advanced disease were more prone to have ctDNA detected in plasma, as evidenced by higher tumor burden (*p* = 0.005), involvement of several anatomic sites (*p* = 0.009), and having received several lines of TKIs (*p* = 0.003) (Additional file 5: Table S5).

**Fig. 3:**
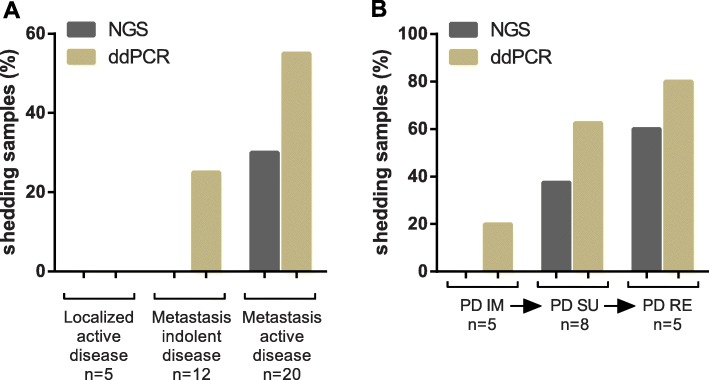
Percentage of shedding samples by ddPCR and NGS according to disease status (localized/metastatic and indolent/active disease) (**a**) and the line of treatment for metastatic disease (**b**). Indolent disease refers to stable disease at the time of blood draw, while active disease samples were collected in progressing patients at the time of blood draw

ctDNA could not be detected in localized GIST with NGS, nor with ddPCR (0/5, 0%). Detection rate increases in metastatic patients (38.5%, 5/13), and particularly at the time of active/progression disease in imatinib-resistant patients. The highly-sensitive ddPCR technology raised the ratio of ctDNA detection, being also capable to detect known cfDNA mutations in one patient at the time of imatinib progression, and in a small proportion of cases with indolent disease (responsive to treatment) (Fig. 3a and b). Thus, although ctDNA detection in plasma of GIST patients is overall low, both NGS and ddPCR appear to detect cfDNA mutations more efficiently in GIST patients with progressive disease and after one or more lines of treatment.

### Serial monitoring of mutated plasma cfDNA in metastatic GIST patients

**Fig. 4:**
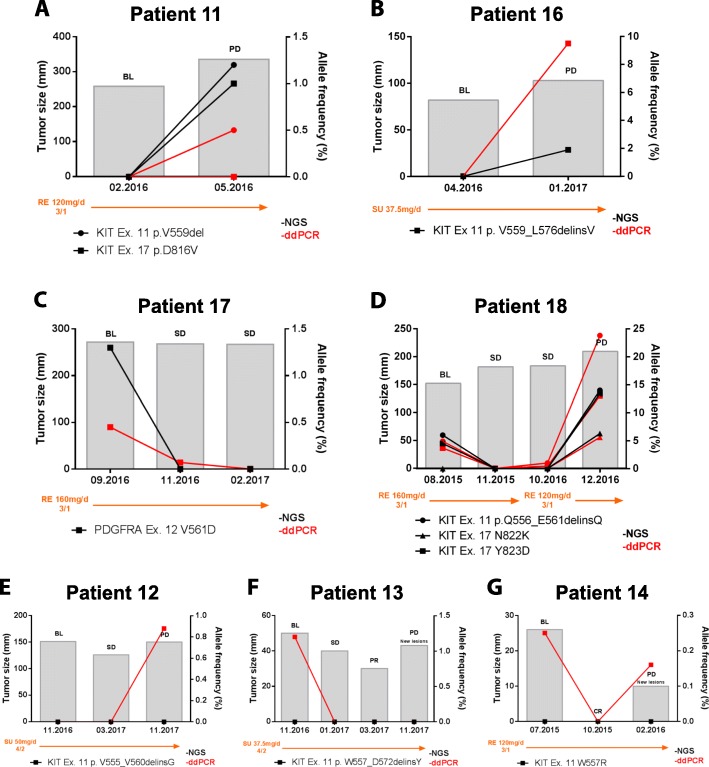
ctDNA monitoring. Patient-specific ctDNA profiles from cohort B (serial plasma monitoring) characterized by NGS and confirmed by ddPCR. The levels of ctDNA varies according to the clinical course throughout the treatment. Black and red lines denote corresponding cfDNA mutations detected by NGS and ddPCR, respectively. Gray columns denote tumor burden in millimeters, and has been calculated according to RECIST criteria. Specific treatment, treatment modifications, and dose, are provided below each graph. ctDNA was detected in patients 11, 16, 17 and 18 by both NGS and ddPCR (**A**-**D**), and only by ddPCR in patients 12, 13 and 14 (**E**-**G**). Two patients were NGS and ddPCR silent and are not shown in this Fig. BL, baseline; PD, progression disease; SD, stable disease; PR, partial response; CR, complete response; SU, sunitinib; RE, regorafenib

From nine metastatic, imatinib-resistant GIST patients treated with sunitinib or regorafenib and with serial plasma determinations (cohort B - serial), both NGS and ddPCR found cfDNA mutations in four patients (44%), whereas ddPCR detected ctDNA in three NGS-silent patients. Two patients were NGS- and ddPCR-silent (Fig. 4). NGS of plasma, when positive, largely paralleled tumor evolution (Fig. 4a-d). Thus, patient 11 disease progression after 2 months of regorafenib evidenced in plasma the emergence of the multi-resistant *KIT* exon 11 D816V mutation [30] that was present in tumor tissue prior to the initiation of the therapy. Similarly, sunitinib disease progression in patient 16 and stabilization in patient 17 on regorafenib were paralleled, respectively, by increase in primary *KIT* exon 11, and suppression of primary *PDGFRA* exon 12 mutation, although no secondary clones were detected. Clonal dynamics were particularly interesting in patient 18, in which standard dose of regorafenib (160 mg daily, 3 weeks on, 1 week off) led to disease stabilization together with suppression of primary *KIT* exon 11 and secondary *KIT* exon 17 Y823D mutations to undetectable levels for more than 1 year. Disease progression occurred 2 months after a necessary dose reduction, accompanied by re-emergence of *KIT* exon 17 Y823D resistant mutation and the appearance of a new *KIT* exon 17 N822K clone, likely due to insufficient dose of regorafenib against KIT secondary mutations. Detection of these mutations was largely confirmed by respective ddPCR assays. Remarkably, a slight increase in the AF of the primary *KIT* exon 11 mutation (1%) and the secondary *KIT* exon 17 Y823D (0,45%) was observed by ddPCR on the third timepoint (10.2016) before radiological tumor progression, thereby anticipating in 2 months the eventual disease progression. Together, detection and monitoring of primary and secondary resistance mutations in ctDNA appears to provide dynamic information in a subset of imatinib-resistant GIST patients.

Known KIT primary mutations could be followed by ddPCR through serial plasma determinations in three of five NGS-silent patients (Fig. 4e-g). Although AFs were mostly low (<1%), determination of known KIT primary mutations reproduced to a large extent the clinical course of disease in these three patients.

### Mechanisms of resistance after imatinib failure

Our series included tumor and plasma samples from 10 imatinib-resistant GIST patients progressing to sunitinib or regorafenib (Fig. 2; Additional file 4: Table S4). Three different resistance mutations involving KIT activation-loop were identified by NGS of plasma in two patients (Fig. 4a and d). In the remaining patients with negative ctDNA studies for resistance mutations, two patients (9 and 10) harbored four imatinib-resistance mutations in tumor tissue, also affecting KIT activation-loop (Table 2), while three patients had progressing lesions associated with phenotype change – namely, shift towards epithelioid and pleomorphic features together with loss or substantial decrease in KIT expression -, but no resistance mutations emerged from NGS of tumor tissue (Additional file 7: Figure S2). Additional file 8: Figure S3 summarizes these findings. Collectively, ctDNA and tissue sequencing data suggest that other mechanisms beyond KIT secondary mutations may arise after several lines of treatment with KIT inhibitory therapies.

## Discussion

ctDNA evaluation might impact particularly in tumor types associated with recurrent driver genetic events, such as GIST, a rare neoplasm of mesenchymal origin whose course of disease is governed by KIT or PDGFRA oncogenic activation [5]. The diversity of primary and secondary mutations across well-known exonic regions of the *KIT* gene [3–6, 8–10, 12, 16] positions NGS as a more suitable technology than digital PCR for exhaustive evaluation of driver and resistance mutations in plasma. Nonetheless, strict criteria must be followed prior to the implementation of liquid biopsy into the clinic [28].

We investigated the validity and utility of amplicon-based plasma NGS to detect molecular alterations in GIST patients. To this purpose, matched tumor tissue was collected at the time of plasma sampling or at diagnosis and served as the reference standard. ddPCR was used for orthogonal validation of mutations found by NGS in both tumor tissue and plasma. Our amplicon pipeline was also improved for the detection of long indels in KIT, which is a common challenge across NGS-based technologies. The overall sensitivity for detection of tumor tissue mutations in cfDNA was 28.6%, showing high concordance with ddPCR in the confirmation analyses. No KIT or PDGFRA primary mutations were detected in the 5 localized GIST. Conversely, amplicon-sequencing detected cfDNA mutations in 38% metastatic GIST patients. Few case series and reports have addressed the role of ctDNA in GIST. This evidence shows that mutant KIT and PDGFRA can be detected and quantified in plasma of GIST patients, also with a predilection for patients with high tumor burden [35–37]. However, our results differ in some regards from these studies. Prior NGS-based analyses detected ctDNA in 17–70% localized, and in 100% metastatic GIST patients [36–39]. Conversely, NGS detection rate in our population was lower. Even the more sensitive and specific ddPCR technology only reached an overall sensitivity of 42.9% in our series, failing to detect mutations in localized GIST. Several factors may have accounted for these disparities. First, we applied a stringent criteria for NGS variant calling AF at ≥1%, also increasing the average coverage for plasma samples to 5000x. Consequently, we validated that our panel was robust to detect cfDNA mutations at AFs ≥1%, while most of the discrepancies observed between amplicon sequencing and ddPCR were shown at low AFs. These validations involved orthogonal NGS of tumor tissue, as the reference standard, and plasma variants cross-validation with ddPCR, thus following recent ASCO Guidelines recommendations [28]. Second, prior NGS studies in GIST did not incorporate variant calling algorithms for variants at low AF (< 5%) and mostly relied on manual inspection of raw data based on mutational findings in tumor tissue. This method is biased, since it enhances ctDNA detection. Moreover, ctDNA findings were not validated with a different technology. Therefore, this approach, although feasible, lacks clinical utility because it is not systematic to be implemented in the routine clinical care. Third, there is no established optimal lower limit of detection of ctDNA, and it varies depending on each assay and its intended use. Nonetheless, several studies have recently shown that the lower the variant AFs (< 1%), the lower the concordance between plasma and tissue genotyping and the higher the rate of discrepancies among NGS platforms [24, 40, 41].

ctDNA was found in a low proportion of GIST patients (27.8%) and at low AF (6.2%, range 1–14%) compared to the majority of neoplasms [21–24, 42]. These findings are unexpected since the bulk of disease in metastatic GIST patients is usually higher than in other cancer types, as reflected by a median tumor burden of 15.2 cm in our series. Accordingly, prior data in GIST have also reported low AF of mutations found in plasma, which is also in line with the scarce works studying ctDNA in sarcomas [42, 43] and further supports the sensitivity reached by NGS in our series. Thus, this collective evidence indicates that ctDNA shedding appears to be low in malignant mesenchymal neoplasms. Although inter-studies comparisons are challenging, the proportion of metastatic GIST patients with ctDNA detected by NGS or ddPCR lies in the medium-to-low range compared with other epithelial neoplasms analyzed with several high sensitive techniques, including NGS [21]. Thus, intrinsic GIST biological characteristics might condition a lower ctDNA shedding than expected.

We found amplicon sequencing of ctDNA informative in a subset of GIST, mainly in metastatic, progressive disease after imatinib failure. Therefore it has the potential to avoid tumor biopsies when tumor genotyping is required. Additionally, serial ctDNA assessment reproduces the course of the disease and provides information on subclonal dynamics. Notably, we confirmed that monitoring of known KIT or PDGFRA mutations in plasma with ddPCR is useful in a bigger subset of GIST patients, and that might predict tumor progression before radiological evaluation. Nonetheless, NGS of plasma advantages digital PCR-based technologies in the detection of the higher variety of mutations found in GIST. This is non-trivial, since KIT secondary genotype predicts response to TKIs after imatinib failure [15–17, 30], and therefore, serial plasma determination of cfDNA mutations help to guide treatment decisions in GIST patients. For instance, NGS of plasma in patient 18 adds further evidence supporting that regorafenib is predominantly active against secondary mutations in the activation loop [15, 30], and suggests that regorafenib dose is critical for the effective suppression of resistant subclones.

Unlike prior reports, an important focus of our studies was on imatinib-resistance disease, with 11 out of 18 patients in this setting. We did not identify substantial heterogeneity of KIT secondary mutations neither in plasma nor in tumor tissue, which agrees with previous PCR-based studies [9, 10, 16, 44] and more recent plasma NGS reports [36, 37, 39]. Likewise, we did not observe either enrichment in KIT-downstream molecules as a resistance mechanism to TKIs with KIT inhibitory activity [11], although phenotypical changes were shown in three patients with matched NGS of tissue and plasma. This, in turn, highlights unknown KIT-independent underlying mechanisms of resistance not captured with the NGS panel. Thus, the only mechanism of resistance identified in our series in GIST patients progressing to sunitinib or regorafenib consisted on secondary mutations in the KIT activation loop. These data will need to be verified in further series.

The main limitation from our study is the cohort size: despite exhaustive inter-platform analysis and cross-validations, these low numbers cannot capture the biological complexity of this disease from paucisymptomatic localized tumors to TKI-refractory disease. This limitation, also affecting prior publications in GIST and sarcomas [35, 36, 38, 39, 42, 43, 45–47], would benefit from international consortiums delving deeper the clinical utility of ctDNA in malignant mesenchymal neoplasms. Likewise, the role of other circulating markers [48] or epigenetic biomarkers [49] with potential role in tumor diagnosis, monitoring and response evaluation is yet to be defined in GIST.

Novel ultra-deep NGS assays for plasma sequencing have the potential to detect a wider array of mutations at lower AFs, and therefore, to provide more thorough information regarding monitoring and determination of resistance mechanisms. However, the aforementioned challenges with variants at low AF (< 1%) are yet to be technically addressed in the forthcoming years [24, 40, 41], particularly in a disease like GIST with low ctDNA shedding and AF. Amplicon sequencing of plasma with high coverage (5000x), when correctly validated for detection of cfDNA mutations, has the advantages to detect robustly plasma mutations at AFs ≥ 1%, less expensively, and with the potential to be successfully implemented in a higher number of oncology centers with expertise in NGS, given the widespread use of amplicon-based NGS platforms in molecular prescreening programs. Although likely less sensitive than other approaches, recent studies support its use for ctDNA determination [26, 27].

## Conclusions

Amplicon-based NGS robustly detects cfDNA mutations in a subset of GIST patients, mostly restricted to imatinib-resistance, progressive and bulky disease. Although ddPCR is more sensitive than NGS, overall, ctDNA levels in GIST appear to be lower in GIST than in other neoplasms, which limits its clinical use.

## Supplementary information

**Additional file 1: Table S1**. Genes covered by VHIO amplicon-sequencing panel.

**Additional file 2: Table S2**. Primers and sequences for ddPCR.

**Additional file 3: Table S3**. Known *KIT* exon 11 long/complex indels called with two different pipelines.

**Additional file 4: Table S4.** Correlation of KIT/PDGFRA genotype between tissue and plasma.

**Additional file 5: Table S5**. Association between clinicopathological factors and the presence of ctDNA in plasma.

**Additional file 6: Figure S6.** Concordance of allele frequency between NGS and ddPCR for detection of plasma mutations in all plasma samples studied with both assays (A). ctDNA detection and allele frequencies distributed by samples detected only by ddPCR, NGS or both technologies.

**Additional file 7: Figure S7.** Hematoxylin & eosin and c-KIT immunohistochemical stains for GIST cases 8 (A, B), 12 (C, D) and 10 (E, F) at baseline (A, C, E) and at the time of tumor progression (B, D, F), showing loss of c-KIT expression in the absence of resistance mutations.

**Additional file 8: Figure S8.** Schematic view of the resistance mechanisms found in our series by NGS of plasma and tissue, and histological evaluation. Mechanisms of resistance are grouped according to its determination by ctDNA evaluation or by tumor tissue sequencing or histological evaluation.

## Data Availability

All data generated or analyzed during this study are included in this published article and its supplementary information files.

## Abbreviations

AF: allele fraction
cfDNA: cell free DNA
ctDNA: circulating tumor DNA
ddPCR: droplet digital PCR
FFPE: formalin fixed, paraffin-embedded
GIST: gastrointestinal stromal tumor
NGS: next generation sequencing
RTK: receptor tyrosine kinase
VAF: variant allele frequency
