# Supplementary figures and images for: Clinical value of next generation sequencing of plasma cell-free DNA in gastrointestinal stromal tumors
Source: BMC Cancer. 2020 Feb 5;20:99. doi: 10.1186/s12885-020-6597-x (PMC7003348; doi:10.1186/s12885-020-6597-x)

Supplementary material

Supplementary Figure 1

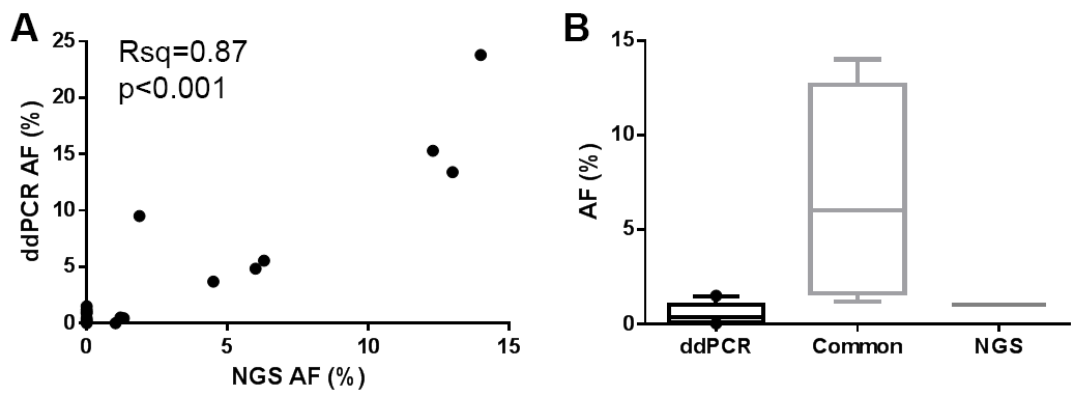

Supplement: Supplementary file 1 — Additional file 1: Table S1. Genes covered by VHIO amplicon-sequencing panel. [file 12885_2020_6597_MOESM1_ESM.pdf]

Supplementary Figure 2

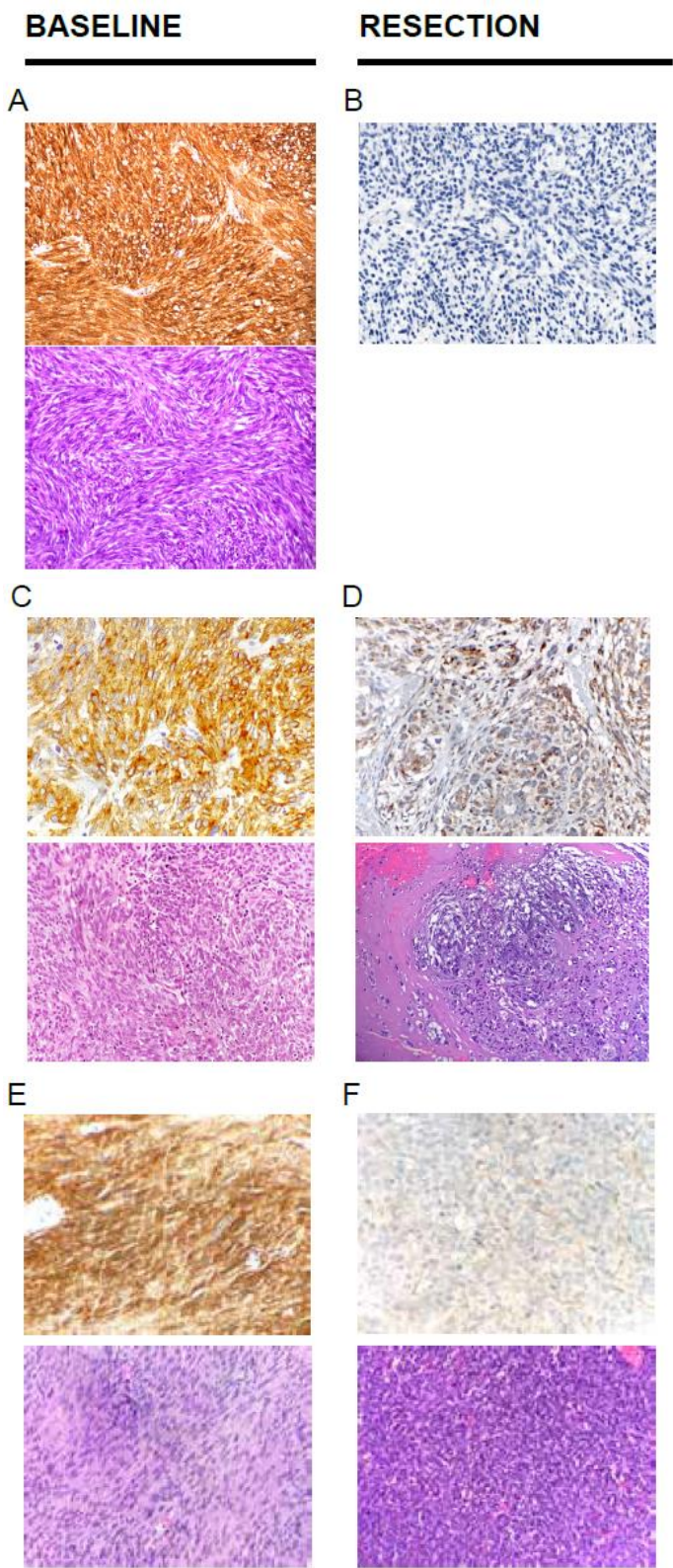

Supplement: Supplementary file 2 — Additional file 2: Table S2. Primers and sequences for ddPCR. [file 12885_2020_6597_MOESM2_ESM.pdf]

Supplementary Figure 3

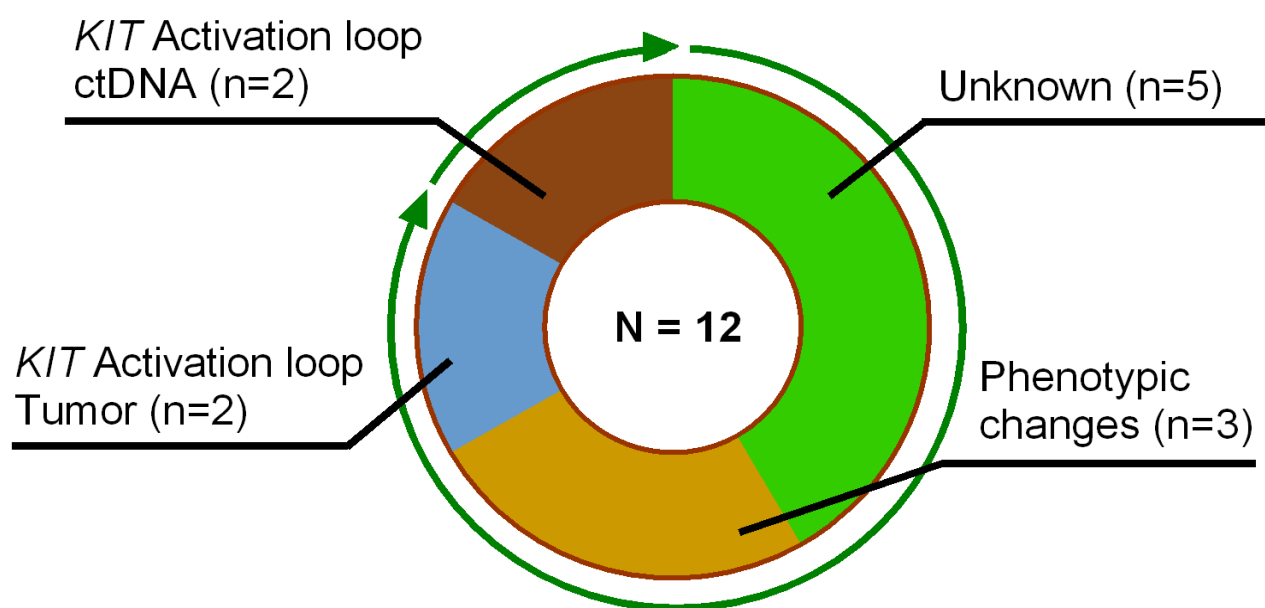

Supplement: Supplementary file 3 — Additional file 3: Table S3. Known KIT exon 11 long/complex indels called with two different pipelines. [file 12885_2020_6597_MOESM3_ESM.pdf]
