# Supplementary material for: Clinical value of next generation sequencing of plasma cell-free DNA in gastrointestinal stromal tumors
Source: BMC Cancer. 2020 Feb 5;20:99. doi: 10.1186/s12885-020-6597-x (PMC7003348; doi:10.1186/s12885-020-6597-x)
Supplement: Supplementary file 4 — Additional file 4: Table S4. Correlation of KIT/PDGFRA genotype between tissue and plasma. [file 12885_2020_6597_MOESM4_ESM.docx]

**Additional file 4:Table S1**. Genes covered by VHIO amplicon-sequencing panel.

| ABL1 | ERBB2 | HRAS | MSH6 | PTEN |
| --- | --- | --- | --- | --- |
| AKT1 | ERBB3 | IDH1 | MTOR | RB1 |
| AKT2 | ESR1 | IDH2 | MYC | RET |
| AKT3 | FBXW7 | JAK1 | NF1 | RUNX1 |
| ALK | FGFR1 | JAK3 | NF2 | SMAD4 |
| APC | FGFR2 | KIT | NOTCH1 | MARCB1 |
| BRAF | FGFR3 | KRAS | NOTCH4 | SRC |
| CDH1 | FLT3 | MAG | NRAS | STK11 |
| CDKN2A | GATA1 | MAP2K1 | PDGFRA | TP52 |
| CSF1R | GNA11 | MET | PIK3CA | TSC1 |
| CTNNB1 | GNAQ | MLH1 | PIK3R1 | TSC2 |
| EGFR | GNAS | MPL | PIK3R5 | VHL |
