# Supplementary material for: Clinical value of next generation sequencing of plasma cell-free DNA in gastrointestinal stromal tumors
Source: BMC Cancer. 2020 Feb 5;20:99. doi: 10.1186/s12885-020-6597-x (PMC7003348; doi:10.1186/s12885-020-6597-x)
Supplement: Supplementary file 5 — Additional file 5: Table S5. Association between clinicopathological factors and the presence of ctDNA in plasma. [file 12885_2020_6597_MOESM5_ESM.docx]

**Additional file 5: Table S2**. Primers and sequences for ddPCR.

| **Assays** | **Forward Primer Sequence 5'->3'** | **Reverse Primer Sequence 5'->3'** | **Mutant Probe 5'->3'** | **Wild Type Probe 5'->3'** |
| --- | --- | --- | --- | --- |
| ***KIT Ex 11 p.K550_K558del*** | AGGTGATCTATTTTTCCCTTTCTCC | AAGGAAGTTGTGTTGGGTCTATGTAA | CACAGGTTGTTGAGGAG | CCCATGTATGAAGTACA |
| ***KIT Ex 11 p.M552_E554delinsK*** | AATAATTATTAAAAGGTGATCTATTTTTCCCTTTCTCC | CCATTTATCTCCTCAACAACCTTCCA | ACAGAAACCCAAAGTAC | CCACAGAAACCCATGTATG |
| ***KIT Ex11 Q556_E561delinsQ*** | TTCCCTTTCTCCCCACAGAA | ACTCCCATTTGTGATCATAAGGAAG | GTATGAAGTACAGGAGATAA | TATGAAGTACAGTGGAAGGT |
| ***KIT Ex 11 p.V555_K558del*** | TTCCCTTTCTCCCCACAGAA | GGAAGTTGTGTTGGGTCTATGTAAAC | TGAAGTTGTTGAGGAGAT | CCATGTATGAAGTACAGTGG |
| ***KIT Ex 11 p.V555_V560delinsG*** | TTCCCTTTCTCCCCACAGAA | GATCATAAGGAAGTTGTGTTGGGTC | TGAGGGTGAGGAGATA | GAAGTACAGTGGAAGGT |
| ***KIT Ex 11 p.Q556_K558delinsQ*** | CTCCCCACAGAAACCCATGT | GGAAGTTGTGTTGGGTCTATGTAAACATA | CTCAACAACCTGTACTTC | CCTTCCACTGTACTTCAT |
| ***KIT Ex11 Q556_E561delinsQ*** | TTCCCTTTCTCCCCACAGAA | ACTCCCATTTGTGATCATAAGGAAG | GTATGAAGTACAGGAGATAA | TATGAAGTACAGTGGAAGGT |
| ***KIT Ex 11 p.W557R*** | CCCCACAGAAACCCATGTATGAA | TGTTGGGTCTATGTAAACATAATTGTTTCC | ACAACCTTCCGCTGTAC | CAACAACCTTCCACTGTAC |
| ***KIT Ex 11 p.W557_K558delinsFP*** | CCCCACAGAAACCCATGTATGAAG | TTGTGTTGGGTCTATGTAAACATAATTGTT | ACAACCGGAAACTGTA | CTCAACAACCTTCCACTGTA |
| ***KIT Ex 11 p.W557_D572delinsY*** | TTCCCTTTCTCCCCACAGAA | AACTCAGCCTGTTTCTGGGAAA | TACAGTACCCAACACAAC | GGTTGTTGAGGAGATAA |
| ***KIT Ex 11 p.V559del*** | CCCACAGAAACCCATGTATGAAGTA | AGGAAGTTGTGTTGGGTCTATGTAAAC | TCTCCTCAACCTTCCAC | TCCTCAACAACCTTCCAC |
| ***KIT Ex 11 p.V559_L576delinsV*** | TCCCTTTCTCCCCACAGAAAC | AACTCAGCCTGTTTCTGGGAAAC | AAGGTTCCTTATGATCAC | TTGAGGAGATAAATGG |
| ***KIT Ex 11 p.Y568_L576delinsCV*** | AAGGTTGTTGAGGAGATAAATGGAA | AACTCAGCCTGTTTCTGGGAAA | CAATTGTGTTCCTTATGAT | ATGTTTACATAGACCCAACA |
| ***KIT Ex 11 p.L576P*** | TTGTTGAGGAGATAAATGGAAACAATTATGT | AAACTCAGCCTGTTTCTGGGAAA | CCAACACAACCTCCTTAT | CCCAACACAACTTCCTTAT |
| ***KIT Ex 17 p.D816V*** | CTCATGGTCGGATCACAAAGATTTG | GGGTACTCACGTTTCCTTTAACCA | TAGCCAGAGTCATCAAG | TAGCCAGAGACATCAAG |
| ***KIT Ex 17 p.N822K*** | ATTTGTGATTTTGGTCTAGCCAGAGA | GCAGAGAATGGGTACTCACGTTT | CCTTTAACCACATACTTAGAA | CCTTTAACCACATAATTAGAA |
| ***KIT Ex 17 p.N822K*** | ATTTGTGATTTTGGTCTAGCCAGAGA | GCAGAGAATGGGTACTCACGTTT | CCTTTAACCACATATTTAGAA | CCTTTAACCACATAATTAGAA |
| ***KIT Ex 17 p.Y823D*** | ATTTGTGATTTTGGTCTAGCCAGAGA | GCAGAGAATGGGTACTCACGTTT | CCTTTAACCACATCATTAGA | CCTTTAACCACATAATTAGA |
| ***PDGFRA Ex 12 p.V561D*** | TCCTGGTCATTTATAGAAACCGAGGTA | CGGGTCCACATAAATATATTCATGTCCAT | TTGATTCAATGTCCCTCC | ATTGATTCAATGACCCTCC |
| ***PDGFRA Ex 18 p.D842V*** | GCAACGTCCTCCTGGCAC | CTGCCTTTCGACACATAGTTCG | CAGAGTCATCATGCATGA | AGAGACATCATGCATGAT |
| ***PDGFRA Ex 18 p.D842_D846delinsN*** | CAAGGAAAAATTGTGAAGATCTGTGA | CCTGACCAGTGAGGGAAGTGAG | TGGCCAGAAATT | GCCAGAGACATCAT |
