# Supplementary material for: Clinical value of next generation sequencing of plasma cell-free DNA in gastrointestinal stromal tumors
Source: BMC Cancer. 2020 Feb 5;20:99. doi: 10.1186/s12885-020-6597-x (PMC7003348; doi:10.1186/s12885-020-6597-x)
Supplement: Supplementary file 6 — Additional file 6: Figure S6. Concordance of allele frequency between NGS and ddPCR for detection of plasma mutations in all plasma samples studied with both assays (A). ctDNA detection and allele frequencies distributed by samples detected only by ddPCR, NGS or both technologies. [file 12885_2020_6597_MOESM6_ESM.docx]

**Additional file 6: Table S3**. Known *KIT* exon 11 long/complex indels called with two different pipelines.

| **Case ID** | **Origin** | **Sanger Sequencing**  **KIT Ex. 11^a^** | **Standard Pipeline AF** | **Indel Pipeline AF** | **Amplicon-seq interpretation** |
| --- | --- | --- | --- | --- | --- |
| **GIST-T1** | Cell line | V560_Y578del | WT | 50.0% | c.1679_1735del:p.V560_Y578del |
| **GIST430** | Cell line | V560_L576del | WT | 19.4% | c.1678_1728del:p.V560_L576del |
| **192** | FFPE | dup570-579 | WT | 8.6% | c.1713_1739dup:p.D579_H580insQDPTQLPYD |
| **142** | FFPE | del552-559 | 73.8% | 57.6% | c.1654_1677del:p.M552_V559del |
| **153** | FFPE | del567-573 | 27.2% | 26.5% | c.1676T>A:p.V559D |
| **498** | FFPE | L589L+dup575-589 | WT | 6.5% | c.1729_1730insCTTATGATCACAAATGGGAGTTTCCCAGAAAC  AGGCCACAACTTC:p.R588_L589insPQLPYDHKWEFPRNR |
| **A2** | FFPE | dup573-591 | WT | 0.3% | c.1775_1776insTCCAACACAACTTCCTTATGATCACAAATGGG  AGTTTCCCAGAAACAGGCTGAGTTTTGG:p.P573_G592dup |
| **R9** | FFPE | del550-560 | 56.0% | 47.2% | c.1661_1678del:p.E554_V559del |
| **A1** | FFPE | del558-572 | WT | WT | WT |

^a^Sanger sequencing mutations is shown with the nomenclature available as recorded in the database.

AF: allele frequency, indel: insertion and/or deletion; WT: wild-type; FFPE: formalin-fixed paraffin-embedded.
