# Supplementary material for: Clinical value of next generation sequencing of plasma cell-free DNA in gastrointestinal stromal tumors
Source: BMC Cancer. 2020 Feb 5;20:99. doi: 10.1186/s12885-020-6597-x (PMC7003348; doi:10.1186/s12885-020-6597-x)
Supplement: Supplementary file 7 — Additional file 7: Figure S7. Hematoxylin & eosin and c-KIT immunohistochemical stains for GIST cases 8 (A, B), 12 (C, D) and 10 (E, F) at baseline (A, C, E) and at the time of tumor progression (B, D, F), showing loss of c-KIT expression in the absence of resistance mutations. [file 12885_2020_6597_MOESM7_ESM.docx]

**Additional file 7: Table S4.** Correlation of KIT/PDGFRA genotype between tissue and plasma.

| **Patient ID** | **Cohort** | **Tissue genotype** | **Tumor extension** | **Prim. T. location** | **Mitotic count (/50HPF)** | **Tumor burden (mm)** | **N. organs involved** | **Line of treatment** |
| --- | --- | --- | --- | --- | --- | --- | --- | --- |
| 1 | A | KIT W557R | Localized | Gastric | 4 | 30 | 1 | Naïve |
| 2 | A | KIT L576P | Localized | Jejunum | 3 | 48 | 1 | Naïve |
| 3 | A | PDGFRA D842V | Localized | Gastric | 2 | 120 | 1 | Naïve |
| 4 | A | PDGFRA D842_D846 | Localized | Gastric | 8 | 132 | 1 | Naïve |
| 5 | A | PDGFRA V561D | Localized | Gastric | 0 | 42 | 1 | Naïve |
| 6 | A | KIT Y568_L576 | Metastatic | Jejunum | 44 | 200 | 2 | Naïve |
| 7 | A | KIT K550_K558 | Metastatic | Gastric | 18 | 214 | 2 | Naïve |
| 8 | A | KIT Q556_K558 | Metastatic | Jejunum | 54 | 289 | 2 | Regorafenib |
| 9 | A | KIT W557_K558 + N822K | Metastatic | Gastric | 9 | 28 | 3 | Imatinib |
| 10 | A + B | KIT M552_E554 + D820Y | Metastatic | Rectum | 7 | 82 | 1 | Regorafenib |
| 11 | A + B | KIT V559del + D816V | Metastatic | Ileum | N.A. | 258 | 3 | Sunitinib |
| 12 | A + B | KIT V555_V560 | Metastatic | Rectum | 2 | 151 | 2 | Sunitinib |
| 13 | A + B | KIT T557_D572 | Metastatic | Jejunum | N.A. | 50 | 1 | Sunitinib |
| 14 | B | KIT p.W557R | Metastatic | Gastric | 8 | 26 | 1 | Regorafenib |
| 15 | B | KIT V555_K558 | Metastatic | Unknown | N.E. | 216 | 1 | Sunitinib |
| 16 | B | KIT V559_L576 | Metastatic | Jejunum | 23 | 82 | 2 | Sunitinib |
| 17 | B | PDGFRA V561D | Metastatic | Gastric | N.E. | 272 | 2 | Regorafenib |
| 18 | B | KIT Q556_E561 | Metastatic | Duodenum | 1 | 152 | 4 | Regorafenib |

Abbreviations: ID: identification; Prim. T.: primary tumor; HPF: high-power field; N.: number; N.A.: not available; N.E.: not evaluable.
