# Supplementary material for: Clinical value of next generation sequencing of plasma cell-free DNA in gastrointestinal stromal tumors
Source: BMC Cancer. 2020 Feb 5;20:99. doi: 10.1186/s12885-020-6597-x (PMC7003348; doi:10.1186/s12885-020-6597-x)
Supplement: Supplementary file 8 — Additional file 8: Figure S8. Schematic view of the resistance mechanisms found in our series by NGS of plasma and tissue, and histological evaluation. Mechanisms of resistance are grouped according to its determination by ctDNA evaluation or by tumor tissue sequencing or histological evaluation. [file 12885_2020_6597_MOESM8_ESM.docx]

**Additional file 8: Table S5**. Association between clinicopathological factors and the presence of ctDNA in plasma.

| **Feature** | **N** | **ctDNA positive N (%)** | **p-value** |
| --- | --- | --- | --- |
| Age at diagnosis, mean years (±SD) |  |  | 0.076 |
|  | 64.0 ± 11.6 | 13 (86.7%) |  |
|  | 42.5 ± 6.4 | 2 (13.3%) |  |
| Tumor location |  |  | 0.576 |
| Gastric | 8 | 1 (12.5%) |  |
| Extra-gastric | 9 | 3 (33.3%) |  |
| Primary tumor size, mm (±SD) |  |  | 0.549 |
|  | 98.2 ± 50.3 | 13 (76.5%) |  |
|  | 139 ± 99.8 | 4 (23.5%) |  |
| Mitotic count /50HPF, mm (±SD) |  |  | 1.000 |
|  | 10.7 ± 12.5 | 12 (85.7%) |  |
|  | 27.5 ± 34.5 | 2 (14.3%) |  |
| Tumor stage at diagnosis |  |  | 0.092 |
| Localized | 8 | 0 (0%) |  |
| Metastatic | 10 | 4 (40%) |  |
| Organs involved |  |  | 0.009 |
| 1 | 9 | 1 (11%) |  |
| 2 | 6 | 2 (33.3%) |  |
| 3 | 2 | 1 (50%) |  |
| 4 | 1 | 1 (100%) |  |
| Tumor burden, mm (±SD) |  |  | 0.005 |
|  | 101.5 ± 70.8 | 14 (77.8%) |  |
|  | 242.6 ± 61.8 | 4 (22.2%) |  |
| Primary mutation |  |  | 1.000 |
| KIT | 14 | 3 (21.4%) |  |
| PDGFRA | 4 | 1 (25%) |  |
| Prior lines of treatment |  |  | 0.003 |
| 0 | 7 | 0 (0%) |  |
| 1 | 5 | 0 (0%) |  |
| 2 | 5 | 3 (60%) |  |
| 3 | 1 | 1 (100%) |  |
| cfDNA ng/mL |  |  | 0.271 |
|  | 702.2 ± 731.3 | 32 (86.5%) |  |
|  | 1022.2 ± 854.1 | 5 (13.5%) |  |

N, number; SD, standard deviation; ctDNA, circulating tumor DNA; HPF, high-power fields.
